# Early-Life and Psychosocial Factors in Adults with Symptoms Consistent with Retrograde Cricopharyngeus Dysfunction

**DOI:** 10.3390/jcm15072728

**Published:** 2026-04-04

**Authors:** Jason N. Chen, Cassidy Swain, Duke Appiah, Charles W. Randall, Sandeep Patel

**Affiliations:** 1 Department of Internal Medicine, The University of Texas Health Science Center at San Antonio, San Antonio, TX 78229, USA; 2 Department of Public Health, Texas Tech University Health Sciences Center, Lubbock, TX 79430, USA; 3 Gastroenterology Research of America, San Antonio, TX 78229, USA; 4 Department of Gastroenterology, University Health System, San Antonio, TX 78229, USA

**Keywords:** motility disorders, nerve-gut interactions, esophageal motility disorder, esophageal sphincter, genetics

## Abstract

**Background**: Retrograde cricopharyngeus dysfunction (RCPD) is a recently described upper esophageal sphincter motility disorder caused by the inability of the cricopharyngeus muscle to relax, prohibiting belching. While clinical features and treatment have been reported, early-life experiences remain unclear. This study aimed to explore childhood experiences, comorbidities, and family history in adults reporting symptoms consistent with RCPD. **Methods**: This cross-sectional survey included adults recruited through an online community focused on RCPD who reported cardinal symptoms consistent with RCPD. The survey collected and descriptively analyzed demographics, symptom profile, family history, neonatal and childhood experiences, psychological factors, and physician visits. **Results**: Of 225 respondents, 207 met inclusion criteria (mean age 32 years; 69% female). Nearly all experienced abdominal bloating (98%), gurgling noises (98%), flatulence (90%), and inability to belch (100%). Painful hiccupping, a newer described symptom, was reported by 80%. Symptoms began before age 25 in 97%, and 29% reported a first-degree relative affected. Common early-life experiences included emetophobia (39%), anxiety (38%), and difficulty being burped as an infant (20%). No statistically significant crude differences were detected in symptom severity, frequency, gender, or age of onset by presence of experiences. Only 36% felt that any physician understood their condition, and 18% reported their gastroenterologist improved their symptoms. **Conclusions**: Psychological early-life experiences and family history were common, but exploratory analyses did not detect statistically significant differences in symptom burden by their presence. These findings provide a foundation for future studies investigating the disorder’s pathophysiology. Limited physician recognition highlights the need for greater clinical awareness of this emerging esophageal motility disorder.

## 1. Introduction

Retrograde Cricopharyngeus Dysfunction (RCPD) is a relatively newly termed condition that describes the inability of the cricopharyngeus muscle of the upper esophageal sphincter (UES) to relax to allow for burping. Commonly associated symptoms include abdominal bloating, discomfort, excessive flatulence, and socially awkward gurgling noises [1,2]. In addition to the physical discomfort, these symptoms can significantly affect an individual’s quality of life, causing social embarrassment and psychological distress [1]. Interviews of those affected have further shown the significant impediment to quality of life, especially due to lack of clinician awareness [3]. Therefore, it is important to continue increasing awareness of this novel condition to improve recognition and management.

While the clinical manifestations of RCPD have been explored, its early-life features remain poorly understood. Previous studies have shown that the vast majority of patients with the condition developed symptoms during early childhood or have lifelong symptoms [1,4]. Although some have family prevalence of the condition [1,4,5], it is uncertain if this is an acquired or inherited condition. It has been noted by Hoesli et al. that some had been colic, gassy, or difficult to burp as an infant [4]. Anecdotally, it has been proposed that some individuals had emetophobia in childhood. It is useful to understand shared early childhood exposures or experiences that may have impacted the rise of this condition.

Many esophageal and gastrointestinal dysfunctions in general have been linked to a variety of demographic, behavioral, and medical factors. For example, conditions like gastroesophageal reflux disease (GERD), esophageal spasms, eosinophilic esophagitis, and achalasia have been associated with smoking, dietary habits, psychological stress, and comorbidities such as allergies and respiratory conditions [6,7,8,9,10]. Neonatal experiences, including feeding difficulties, colic, and premature birth, have also been implicated in early development of esophageal motor dysfunctions [11,12,13,14]. However, the relevance of these factors to RCPD remains unclear.

This study aims to explore common early-life experiences of those who suffer from symptoms consistent with RCPD through a comprehensive survey. Currently, online patient communities focused on RCPD are the source of many previous studies given the novelty of the condition [2,3,15,16,17]. This exploratory, hypothesis-generating study may serve as a foundation for future studies to improve the recognition and management of the condition.

## 2. Methods

This is a cross-sectional exploratory study that utilized a Qualtrics survey that was distributed from December 2024 to February 2025 through an online community focused on RCPD, providing unique access to a concentrated population that is otherwise difficult to identify and recruit through traditional clinical settings. Participation was voluntary and based on convenience sampling within the online community. Adults aged 18–89 years who reported at least one current or prior symptom consistent with RCPD (inability to belch, abdominal bloating/distension, gurgling noises from the chest/lower neck, excessive flatulence, or difficulty vomiting) were invited to participate. These symptoms are commonly used in the clinical diagnosis of RCPD in the current literature [2]. Respondents outside the 18–89 age range, or those who did not endorse at least one cardinal symptom, were excluded from analysis. The survey captured a wide range of variables, including demographics, symptoms experienced, symptom severity and frequency, family history, neonatal and childhood experiences, psychological factors, and physician visits. The complete survey can be found in Supplemental File S1. The questionnaire was developed by the study team to assess clinically relevant domains of interest in RCPD. Early-life and developmental factors examined in this study were selected based on features and comorbidities reported in other esophageal motility and reflux-related disorders [18,19,20,21,22]. To reduce the risk of duplicate or non-independent responses, Qualtrics’ ‘Prevent Multiple Submissions’ feature was enabled, which uses browser cookies to prevent repeat entries from the same device. This study was determined to be exempt from full review by the University of Texas Health San Antonio Institutional Review Board office prior to initiation. This study followed the STROBE reporting guideline for cross-sectional observational studies.

Descriptive analysis, including percentages for categorical variables and means for continuous variables, was conducted through Qualtrics. Proportions for categorical data were calculated out of total respondents for each question. Analyses were performed to compare symptom severity, symptom frequency, gender, and age of onset between participants reporting any early-life experiences versus none, and between participants with versus without a family history of RCPD. Because symptom severity was measured on a scale ranging from 1 to 10, and was not normally distributed, the Wilcoxon rank sum test was used, while chi-square test and Fisher’s exact tests were employed for categorical outcomes of symptom frequency, gender, and age of onset. Statistical significance was determined by *p* values less than 0.05. Analyses were performed using available data for each question; missing responses were excluded on a per-item basis. Consequently, the denominator for each reported percentage reflects the total number of individuals who provided a response to that specific item. All analyses were exploratory, and no correction for multiple testing was applied. All statistical analyses were performed using SAS software version 9.4 (SAS Institute, Inc., Cary, NC, USA).

## 3. Results

### 3.1. Participant Characteristics

Of 225 total respondents, 207 met inclusion criteria for data analysis. Demographic data can be found in Table 1. The average age was 32 years (standard deviation: 9.6). Approximately 27% of respondents were male, while 69% were female. Also, 94% were White, 2% were Asian, 0% were Black, and 5% were another race besides those listed. Regarding ethnicity, 7% reported being Hispanic. A total of 61% of respondents were located in the United States, and 39% were from abroad.

### 3.2. Symptom Profile

Of the respondents, 100% were unable to belch, 98% had abdominal bloating and discomfort, 98% had socially awkward gurgling noises, 90% had excessive flatulence, 80% had painful hiccupping, and 66% had difficulty vomiting (Table 2). In total, 40% noticed symptoms prior to 25 years of age, and 57% have had them for as long as they could remember (Table 2). Prior to treatment, the majority (86%) of respondents had daily symptoms with the mean severity being 7 (standard deviation 1.8) out of 10 (Table 2).

### 3.3. Early-Life Experiences

**Figure 1:**
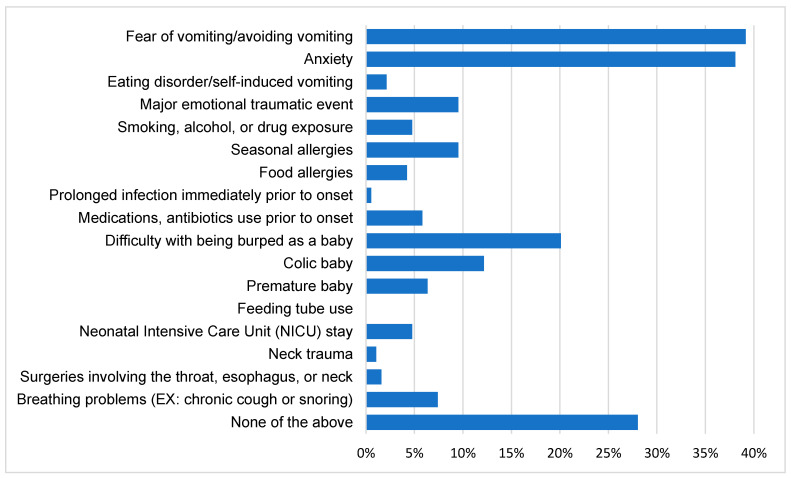
Percentage of respondents experiencing each of the prompts in early life.

Early-life experiences can be found in Figure 1. Of note, given the high proportion of respondents reporting symptoms for as long as they could remember, the temporal relationship between reported early-life factors and symptom onset may not be uniform. Therefore, these factors may reflect antecedents, consequences, or recall-dependent interpretations rather than established early determinants of disease. The most prevalent experiences were emetophobia and anxiety, with approximately 39% and 38% of respondents reporting each respectively. This was followed by difficulty with being burped as a baby at 20%. Other significant factors included 10% reporting having had a major traumatic event, 10% having had seasonal allergies, and 12% having been a colic baby. In total, 28% of respondents exhibited none of the options. In this exploratory analysis, there were no statistically significant differences detected in symptom severity (*p* = 0.93), symptom frequency (*p* = 0.80), gender (*p* = 0.45), or age of onset (*p* = 0.87) between those who selected any of the early-life experiences and none of them (Table 3).

### 3.4. Family History

In total, 29% reported having a family member having the same condition, most commonly a sibling (10%). First-degree relative history accounted for 25% of all respondents and 83% of those who reported having a family member with the condition (Table 2). There were no significant differences detected in symptom severity (*p* = 0.08), symptom frequency (*p* = 0.58), age of onset (*p* = 0.22), or gender (*p* = 0.9) between those who had any family history and those who did not (Table 3).

### 3.5. Comorbidities and Prior Testing

Approximately 26% of respondents had concurrent diagnosed gastroesophageal reflux disease (GERD), 4% had esophageal spasms, 2% had eosinophilic esophagitis, 0% had achalasia, and 73% had none of these (Table 2). There was no statistically significant difference in reported RCPD-like symptom severity between those who reported a diagnosis of GERD (7.3 out of 10) and those who did not (6.9 out of 10) (*p* = 0.16). Of the 94 respondents who were told to take an acid-suppressing medication, 85% did not experience any improvement in symptoms. Additionally, 37% had undergone esophagogastroduodenoscopy (EGD), and 18% had undergone a barium swallow. The most commonly selected treatment option was changing body position, such as laying down.

### 3.6. Physician Encounters and Clinical Recognition

About 61% of respondents reported discussing their condition with any physician (Table 2). Of these, 36% felt that their physician understood how to help them feel better. Also, 36% have seen a gastroenterologist for their symptoms. Of these, 18% felt that their gastroenterologist understood how to help improve symptoms. In total, 32% reported a diagnosis/attribution of their symptoms to GERD rather than RCPD by a physician. Overall, 39% were diagnosed with RCPD by a physician.

## 4. Discussion

This study introduces new evidence of early-life and familial patterns in those with symptoms consistent with RCPD, offering insights for gastroenterologists and other providers confronting this emerging disorder. Because this survey is cross-sectional and descriptive, the findings are presented as prevalence estimates and hypothesis-generating observations rather than demonstrated associations. Like in previous studies, the most common symptoms included the inability to belch, abdominal bloating, socially awkward gurgling noises, and excessive flatulence. However, a less described symptom, painful hiccupping, was described by 80% of participants. This can be seen in other gastric and esophageal conditions that cause irritation along the hiccup reflex arc, which includes the phrenic and vagus nerves [23]. It is unclear if gaseous distention of the esophagus or stomach in RCPD causes a similar pathology.

Also, as in previous studies, symptoms developed early in life, with 97% of participants having developed symptoms by age 25. Previously reported presence of family history of RCPD varies from 17 to 42% [5,24]. In this study, 29% had a family member who had symptoms consistent with RCPD. Of those, 83% were first-degree relatives. Additionally, the presence of family history did not statistically impact symptom severity, frequency, or age of onset. There was also no significant variation in family history prevalence between males and females. The combination of early symptom onset and the prevalence of reported family history raises the hypothesis of a possible inherited contribution that should be further evaluated.

Meanwhile, 72% of respondents related to at least one of the early-life experiences offered in the survey. Despite the high prevalence of several reported early-life experiences, no statistically significant differences were observed in symptom severity, symptom frequency, gender, or age of onset in this exploratory analysis. However, because these analyses were exploratory and based on crude univariable comparisons, the absence of statistically significant differences should not be interpreted as excluding potentially meaningful associations. The most common experiences were psychological, led by fear of vomiting/avoidance of vomiting and anxiety, reported by 39% and 38% of respondents respectively. This is higher than in previous studies, which reported current mental health disorders in 5.7% and 31.8% of RCPD patients [5,16]. The prevalence of anxiety in this study is comparable to a meta-analysis of anxiety prevalence in GERD patients, at 34.4% [25].

There is strong evidence that anxiety and other psychiatric conditions are associated with other esophageal motor dysfunction disorders and greater disease severity, including in GERD, esophageal spasms, and achalasia [25,26,27,28,29,30]. One hypothesis is that psychological stress and hypervigilance may possibly influence upper esophageal sphincter (UES) function via the brain–gut mechanisms; however, this remains speculative and cannot be inferred from descriptive survey data. It has been suggested that anxiety and emetophobia may contribute to esophageal symptoms due to the hypervigilance of esophageal sensation and perception, but may also contribute to physiological changes such as elevated UES basal pressure [31,32,33,34]. It is unknown whether brain–gut interactions or maladaptive learning have any role in RCPD, and this requires evaluation in future studies.

Given the cross-sectional design and that many respondents reported symptoms from as early as they could remember, it cannot be definitively established whether reported experiences preceded symptom onset, developed alongside, or occurred in response to symptoms. Therefore, alternatively, emetophobia may represent a consequence rather than a predisposing factor of RCPD, potentially arising as a response to difficult, painful, or traumatic vomiting experiences related to impaired UES relaxation [35]. Anxiety may also arise secondarily from chronic physical discomfort, social embarrassment, and avoidance behaviors [1], and symptom-specific anxiety/hypervigilance has been shown to relate strongly to esophageal symptom severity in patients evaluated for motility disorders [30]. Currently, there is limited literature examining psychological stress in RCPD.

Beyond the UES, external studies using high-resolution manometry have frequently identified ineffective esophageal motility (IEM) in patients with RCPD symptoms [36]. Whether altered esophageal body motility contributes to gas trapping remains uncertain, as does whether IEM represents a coexisting finding or contributes to symptom severity [36].

It is unclear whether symptom complexes consistent with RCPD are associated with other esophageal pathologies. Overall, 27% reported being diagnosed with either GERD, esophageal spasms, or eosinophilic esophagitis in addition to their RCPD-like symptoms. The most common of these, GERD, existed in 26% of respondents. Of note, because GERD was self-reported, these responses may represent reflux/GERD symptoms or prior clinical labeling rather than objectively confirmed GERD. No one reported having achalasia. Current existing literature, although limited, reveals limited comorbidities in RCPD, but GERD was found in 6.6–50% of patients [1,5,16,37]. There was no statistically significant difference in reported RCPD-like symptom severity between those reporting a concurrent diagnosis of GERD and those without in this study. GERD is known to affect the motility of the esophagus [38]. There is no causal relationship between GERD and RCPD, but it has been hypothesized that chronic reflux exposure to the UES in GERD may cause compensatory hypertrophy of the cricopharyngeus muscle, predisposing to RCPD [39]. However, the possible relationship between this disorder and other examined esophageal disorders is also complicated by their shared confounding associations with stress and anxiety.

This study further reinforces the limited awareness of RCPD or RCPD-like symptom presentations among providers. Of the 61% of respondents who have sought help from a physician, only 36% felt that their physician understood the disease enough to adequately manage it. Furthermore, only 36% of respondents have seen a gastroenterologist, of whom only 18% thought it was helpful. In total, 32% felt that their symptoms were attributed to GERD instead of RCPD. Many claimed inadequate treatment with proton pump inhibitors and had undergone with EGDs, barium swallow studies, computed tomography scans, or gastric emptying studies, in which efficacy for diagnosing RCPD is unclear [16,36], further suggesting a knowledge gap among providers in the diagnosis and treatment of RCPD. It is also important to note that the majority of this population have not been seen by gastroenterologists, who are able to perform esophageal high-resolution manometry (HRM), one of the diagnostic tests for RCPD [36,40]. Additionally, recruitment through an online community may introduce self-selection and self-diagnosis bias, which could overrepresent individuals with more severe symptoms or poorer outcomes. Reports of diagnostic delay and misdiagnosis should therefore be interpreted as patient-perceived experiences rather than clinically confirmed.

Awareness of RCPD appears to involve contributions from both the patient and the provider. From the patient perspective, social media and online forums may play an important role in awareness of the condition. Previous studies have demonstrated that the majority of patients first learn of RCPD through social media or online forums [2,3,15,16,17]. Beyond awareness, this study demonstrates how online patient communities can serve as valuable platforms for recruiting individuals with rare, under-recognized disorders. From the provider perspective, the limited physician recognition reported by participants may reflect incomplete clinical awareness that may contribute to delayed diagnosis. Increasing recognition of symptoms consistent with RCPD among frontline providers could facilitate more timely referral to appropriate specialists, such as gastroenterologists, otolaryngologists, or speech-language pathologists, for further evaluation. Taken together, these findings provide descriptive information about symptom-defined RCPD presentations and generate hypotheses for future study.

## 5. Limitations

This study is limited by its reliance on self-reported survey data that is not physician-verified, leading to potential misclassification. Given the cross-sectional, descriptive design without a control group and limited inferential analyses, findings are best interpreted as prevalence data and hypothesis-generating rather than demonstrating causal or statistically supported associations. While the analysis was restricted to individuals endorsing at least one cardinal symptom of RCPD, many were not officially diagnosed by a clinician, and therefore, the findings are reliant on participant honesty and accuracy. Because participants were recruited through a voluntary online community, the sample may preferentially represent individuals who are more engaged, symptomatic, self-identified, or diagnostically aware than unselected patients in routine clinical care; accordingly, the results of the study may not generalize directly to unselected clinical populations. Because the inability to belch was not graded for completeness or frequency, some respondents may have had partial or intermittent belching, which could introduce heterogeneity and misclassification.

However, given the novelty of this condition and limited consolidated RCPD patient data elsewhere, this study takes advantage of the important education, advocacy, and support role that social media has in health care. Recruitment through an online discussion forum (Reddit) provided unique access to a large, concentrated group of affected patients, as has been utilized previously on smaller scales [1,2,3,15,16,17]. It also captures the perspectives of those who may not have had the opportunity to present to specialty care, a population that is often underrepresented in clinic-based cohorts, especially for novel conditions. Online communities enable rapid recruitment, patient-centered data collection, and unique insights into how individuals experience and discuss their symptoms outside of traditional healthcare environments.

## 6. Conclusions

This study expands the understanding of early-life experiences, family history, and comorbidities reported by adults with self-reported symptoms consistent with RCPD. Anxiety, emetophobia, and early stress exposures were commonly reported among respondents, although their temporal and pathophysiologic relationship to symptoms remains to be clarified. The overlap of commonly reported early-life experiences, psychological distress, and other clinical features may be relevant when considering the overall clinical picture of adults with symptoms consistent with RCPD. At the same time, a substantial proportion of patients reported no identifiable early-life experiences captured by the survey, underscoring the heterogeneity of this condition. Limited physician recognition may delay appropriate management, reinforcing the need for greater clinical awareness and education. Future research could explore potential genetic and neurophysiologic factors to further characterize the development and clinical progression of RCPD.

## Figures and Tables

**Table 1:** Demographic data of respondents.

	*n* (% of Responses for Each Prompt)
Gender (N = 186)	
Identifies as Male	51 (27.4)
Identifies as Female	128 (68.8)
Prefer not to disclose	7 (3.8)
Race (N = 186)	
American Indian or Alaska Native	0 (0.0)
Asian	3 (1.6)
Black or African American	0 (0.0)
Native Hawaiian or Pacific Islander	0 (0.0)
White	174 (93.5)
Other	9 (4.8)
Ethnicity (N = 175)	
Non-Hispanic	162 (92.6)
Hispanic	13 (7.4)
Age (average ± SD)	32.1 ± 9.6
Nationality (N = 199)	
United States	121 (60.8)
Abroad	78 (39.2)

Abbreviations: *n*, number of respondents choosing each response option; N, total number of respondents for the corresponding survey item or item set.

**Table 2:** Overall survey data of respondents.

	*n* (% of Responses for Each Prompt)
Symptoms experienced (N = 207)	
Inability to belch	207 (100.0)
Abdominal bloating or chest pain after eating	203 (98.1)
Socially awkward gurgling noises from the chest and lower neck	202 (97.6)
Excessive flatulence	186 (89.9)
Painful hiccupping	165 (79.7)
Difficulty vomiting	137 (66.2)
Age when first noticed (N = 207)	
5–15	39 (18.8)
16–25	44 (21.3)
26–35	5 (2.4)
36–45	1 (0.5)
46–55	1 (0.5)
56–65	0 (0.0)
66–75	0 (0.0)
I’ve had it for as long as I remember/ I can’t remember ever not having it	117 (56.5)
Frequency of symptoms (N = 194)	
Daily	167 (86.1)
Weekly	21 (10.8)
Monthly	5 (2.6)
Yearly	1 (0.5)
Symptom severity (average ± SD)	7.0 ± 1.8
Family members with the same condition (N = 184)	
Mother	11 (6.0)
Father	12 (6.5)
Sibling	19 (10.3)
Grandparent	2 (1.1)
Cousin	7 (3.8)
Children	4 (2.2)
Other	7 (3.8)
None	130 (70.7)
Other diagnosed conditions (N = 188)	
Achalasia	0 (0.0)
Eosinophilic esophagitis	3 (1.6)
Esophageal spasms	7 (3.7)
Gastroesophageal reflux disease	48 (25.5)
None	138 (73.4)
Felt symptoms were attributed to gastroesophageal reflux disease rather than RCPD (N = 151)	
No	102 (67.5)
Yes	49 (32.5)
Testing performed (N = 190)	
Barium swallow study	35 (18.4)
Computed Tomography scan	22 (11.6)
EndoFLIP	0 (0.0)
Esophagogastroduodenoscopy	71 (37.4)
Gastric emptying study	7 (3.7)
Manometry	12 (6.3)
None	106 (55.8)
If advised to take acid suppressant/ PPI, symptoms improved (N = 94)	
No	80 (85.1)
Yes	14 (14.9)
Methods to resolve symptoms (N = 151)	
Physical activity	13 (8.6)
Change in position	116 (76.8)
Medication	12 (7.9)
Soothing activities	35 (23.2)
Botox injection	39 (25.8)
Other	31 (20.5)
Discussed the condition with a physician (N = 189)	
No	73 (38.6)
Yes	116 (61.4)
Physician understood how to make patient feel better (N = 114)	
No	73 (64.0)
Yes	41 (36.0)
Discussed the condition with a gastroenterologist (N = 187)	
No	119 (63.6)
Yes	68 (36.4)
Gastroenterologist understood how to make patient feel better (N = 68)	
No	56 (82.4)
Yes	12 (17.6)
Formally diagnosed with RCPD (N = 183)	
No	111 (60.7)
Yes	72 (39.3)

Abbreviations: *n*, number of respondents choosing each response option; N, total number of respondents for the corresponding survey item or item set. For multiple-response items, percentages may not sum to 100%.

**Table 3:** Estimates for differences in characteristics according to early-life experience and family history.

Characteristics	Early-life experience		Standardized difference ^1^	Test statistics ^2^ and effect size ^3^	*p* value ^4^
	No (n = 53)	Yes (n = 136)			
Age of onset, n (%)			0.244	χ^2^ = 2.17, V = 0.107	0.874
5–15 years	8 (15.4)	28 (20.4)			
16–25 years	10 (19.2)	31 (22.6)			
26–35 years	1 (1.92)	3 (2.2)			
>35 years	0 (0.0)	2 (1.5)			
For as long as I can remember	33 (63.5)	73 (53.3)			
Gender, n (%)			0.251	χ^2^ = 2.08, V = 0.108	0.445
Identifies as female	37 (77.1)	87 (65.9)			
Identifies as male	10 (20.8)	40 (30.3)			
Prefers not to disclose	1 (2.1)	5 (3.8)			
Symptom frequency, n (%)			0.136	χ^2^ = 1.14, V = 0.078	0.798
Daily	43 (84.3)	118 (86.8)			
Weekly	7 (13.7)	13 (9.6)			
≥Monthly	1 (2.0)	5 (3.7)			
Symptom severity, median (IQR)	7.0 (6.0–8.0)	7.0 (6.0–8.0)	0.007	W = 4652.0, r = 0.006	0.933
Characteristics	Family history		Standardized difference	Test statistics ^1^ and effect size ^2^	*p* value ^3^
	No (n = 130)	Yes (n = 54)			
Age of onset, n (%)			0.413	χ^2^ = 5.81, V = 0.178	0.226
5–15 years	18 (25.9)	14 (13.9)			
16–25 years	28 (22.2)	12 (21.5)			
26–35 years	4 (1.92)	0 (0.0)			
>35 years	1 (0.8)	0 (0.0)			
For as long as I can remember	79 (63.5)	28 (53.3)			
Gender, n (%)			0.093	χ^2^ = 0.26, V = 0.038	0.907
Identifies as female	87(68.0)	38 (71.7)			
Identifies as male	36 (28.1)	13 (24.5)			
Prefers not to disclose	5 (3.9)	2 (3.8)			
Symptom frequency, n (%)			0.055	χ^2^ = 2.67, V = 0.121	0.586
Daily	112 (86.2)	45 (84.9)			
Weekly	14 (10.8)	6 (11.3)			
≥Monthly	2 (3.1)	4 (3.7)			
Symptom severity, median (IQR)	7.0 (6.0–8.0)	7.0 (6.0–8.0)	−0.199	W = 4443.5, r = 0.126	0.088

^1^ Standard difference absolute values ranging from 0.2 to 0.5 are considered small, values of 0.5 to 0.8 are considered medium, and values > 0.8 are considered large differences. ^2^ Test statistics are chi-square test (χ^2^) for categorical variables and Wilcoxon two-sample rank-sum test statistic (W) for continuous variables. ^3^ Effect sizes for categorical variables were measured using Cramér’s V, which assesses the strength of association and ranges from 0 (no relationship) to 1 (complete association), while effect size for continuous variables was Wilcoxon rank-sum *r,* which ranges from −1 to 1, with 0 indicating no difference between groups; |*r*| ≥ 0.1 was considered a small effect, |*r*| ≥ 0.3 a medium effect, and |r| ≥ 0.5 a large effect. ^4^ *p* value for categorical variables based on Fisher’s exact test for categorical variables and Wilcoxon two-sample rank-sum test for continuous variables.

## Data Availability

Deidentified individual participant data will be available to researchers upon request to the corresponding author.

## Supplementary Materials

The following supporting information can be downloaded at https://www.mdpi.com/article/10.3390/jcm15072728/s1, Supplemental File S1: Complete survey.
