# Supplementary material for: Early-Life and Psychosocial Factors in Adults with Symptoms Consistent with Retrograde Cricopharyngeus Dysfunction
Source: J Clin Med. 2026 Apr 4;15(7):2728. doi: 10.3390/jcm15072728 (PMC13074181; doi:10.3390/jcm15072728)
Supplement: Supplementary file 1 [file jcm-15-02728-s001.zip › jcm-4202773-supplementary.pdf]

Supplemental File 1. Complete survey

Dr. Jason Chen, MD at the University of Texas Health and Science Center, will be conducting a research study on the risk factors associated with Retrograde Cricopharyngeus Dysfunction (RCPD). Participating is completely voluntary and participating or not participating in this research study will not affect an individual's academic or professional standing. Willing participants will be asked to complete a 5 minute online survey. There will be no compensation for participating in this study. To protect your confidentiality, survey responses are anonymous, and no personally identifiable information will be asked. As an extra precaution, please do not write in information that could possibly identify you on the open-ended questions. If you have any questions, please reach out to Jason Chen, MD at chenj20@uthscsa.edu.

Q1 Are you aged 18 years or older?

○ Yes

○ No

Q2 Have you ever experienced one of the following conditions? Select all that apply.

☐ The inability to belch

☐ Abdominal bloating and discomfort/nausea, or chest pain, especially after eating

☐ Socially awkward gurgling noises from the chest and lower neck as though the esophagus is churning and straining to eject the air

☐ Excessive flatulence (e.g., farting)

☐ Difficulty vomiting

☐ Painful hiccupping

☐ None

Q3 At around what age did you first notice this issue?

- ○ 5-15
- ○ 16-25
- ○ 26-35
- ○ 36-45
- ○ 46-55
- ○ 56-65
- ○ 66-75
- ○ I've had it for as long as I remember/I can't remember ever not having it

Q4 What factors did you experience/apply to you **before** you developed symptoms? (select all that apply)

- ☐ Fear of vomiting/avoiding vomiting
- ☐ Anxiety
- ☐ Eating disorder/self-induced vomiting
- ☐ Major emotional traumatic event
- ☐ Smoking, alcohol, or drug exposure
- ☐ Seasonal allergies
- ☐ Food allergies
- ☐ Prolonged infection immediately prior to onset
- ☐ Medications, antibiotics use prior to onset
- ☐ Difficulty with being burped as a baby
- ☐ Colic Baby
- ☐ Premature baby
- ☐ Neonatal Intensive Care Unit (NICU) stay as a baby
- ☐ Feeding tube use
- ☐ Neck trauma
- ☐ Surgeries involving the throat, esophagus, or neck
- ☐ Breathing problems (EX: chronic cough or snoring)

- [ ] None of the above

Q5a Did you grow up in the US?

- ◯ Yes
- ◯ No

Q5b If no, where?

____

Q6 How frequently do you experience the conditions you selected previously? If you have received treatment already (eg. botox injection), please indicate frequency before treatment.

- ◯ Yearly
- ◯ Monthly
- ◯ Weekly
- ◯ Daily

Q7 On a scale of 1-10 with 10 being the worst discomfort, how would you rate your discomfort? If you have received treatment already (eg. botox injection), please indicate your discomfort level before treatment.

○ 1

○ 2

○ 3

○ 4

○ 5

○ 6

○ 7

○ 8

○ 9

○ 10

Q8 Have you been diagnosed with one of these conditions? (select all that apply)

▢ Gastroesophageal reflux disease (GERD)

▢ Esophageal spasms

▢ Eosinophilic esophagitis

▢ Achalasia

▢ None of the above

Q9 What, if any, medications do you take for the upper GI tract? (ex: acid suppression like pantoprazole or omeprazole) If none, please skip.

____

Q10 If you were told to take an acid suppressor/proton inhibitor such as pantoprazole or omeprazole for RCPD, did it improve your symptoms? If not applicable, please skip.

○ Yes

○ No

Q11 Have you ever had any testing done? Choose all that apply.

☐ EGD/endoscopy

☐ Barium Swallow

☐ Manometry

☐ CT scan

☐ Endoflip

☐ Gastric emptying study

☐ None of the above

Q12 Please select any of the following strategies you use that **successfully resolve** your symptoms:

- ☐ Physical activity
- ☐ Change of position (e.g., laying down)
- ☐ Medication
- ☐ Soothing activities (e.g., showering, meditation, resting, deep breathing, etc.)
- ☐ Botox injection
- ☐ Other

Q13 Are you aware of others in your family that have the same condition? If yes, select all that apply:

- ☐ Mother
- ☐ Father
- ☐ Sibling
- ☐ Grandparent
- ☐ Cousin
- ☐ Children
- ☐ Other extended family
- ☐ None

Q14 I have discussed this condition with a physician.

- ◯ Yes
- ◯ No

Q16 Specifically, have you seen a gastroenterologist (GI) about your symptoms?

- ◯ Yes
- ◯ No

Q18 Have your symptoms been misdiagnosed as GERD by your physician? (skip if haven't seen a physician)

- ◯ Yes
- ◯ No

Q19 Have you been formally diagnosed with Retrograde Cricopharyngeal Dysfunction (RCPD) by a physician?

- ◯ Yes
- ◯ No

Q20 Please complete the following demographic details (optional)

Q20a Gender

- ◯ Identifies as male
- ◯ Identifies as female
- ◯ Prefers not to disclose

Q20b Current age

____

Q20c Race

- ◯ White
- ◯ Black or African American
- ◯ American Indian or Alaska Native
- ◯ Asian
- ◯ Native Hawaiian or Pacific Islander
- ◯ Other

Q20d Ethnicity

- ◯ Non-Hispanic
- ◯ Hispanic

Q17 Please select the choice that best fits your experience: My gastroenterologist understood how to help me feel better.

- ◯ Yes
- ◯ No

Q15 Please select the choice that best fits your experience: My physician understood how to help me feel better.

- Yes
- No
